# The Prmt5-Vasa module is essential for spermatogenesis in *Bombyx mori*

**DOI:** 10.1371/journal.pgen.1010600

**Published:** 2023-01-12

**Authors:** Xu Yang, Dongbin Chen, Shirui Zheng, Meiyan Yi, Shanshan Wang, Yongjian Liu, Lianyan Jing, Zulian Liu, Dehong Yang, Yujia Liu, Linmeng Tang, James R. Walters, Yongping Huang

**Affiliations:** 1 Key Laboratory of Insect Developmental and Evolutionary Biology, Center for Excellence in Molecular Plant Sciences, Shanghai Institute of Plant Physiology and Ecology, Chinese Academy of Sciences, Shanghai, China; 2 University of Chinese Academy of Sciences, Beijing, China; 3 CAS Center for Excellence in Biotic Interactions, University of Chinese Academy of Sciences, Beijing, China; 4 Department of Ecology & Evolution, University of Kansas, Lawrence, Kansas, United States of America; The University of North Carolina at Chapel Hill, UNITED STATES

## Abstract

In lepidopteran insects, dichotomous spermatogenesis produces eupyrene spermatozoa, which are nucleated, and apyrene spermatozoa, which are anucleated. Both sperm morphs are essential for fertilization, as eupyrene sperm fertilize the egg, and apyrene sperm is necessary for the migration of eupyrene sperm. In *Drosophila*, Prmt5 acts as a type II arginine methyltransferase that catalyzes the symmetrical dimethylation of arginine residues in the RNA helicase Vasa. Prmt5 is critical for the regulation of spermatogenesis, but Vasa is not. To date, functional genetic studies of spermatogenesis in the lepidopteran model *Bombyx mori* has been limited. In this study, we engineered mutations in *BmPrmt5* and *BmVasa* through CRISPR/Cas9-based gene editing. Both *BmPrmt5* and *BmVasa* loss-of-function mutants had similar male and female sterility phenotypes. Through immunofluorescence staining analysis, we found that the morphs of sperm from both *BmPrmt5* and *BmVasa* mutants have severe defects, indicating essential roles for both BmPrmt5 and BmVasa in the regulation of spermatogenesis. Mass spectrometry results identified that R35, R54, and R56 of BmVasa were dimethylated in WT while unmethylated in *BmPrmt5* mutants. RNA-seq analyses indicate that the defects in spermatogenesis in mutants resulted from reduced expression of the spermatogenesis-related genes, including *BmSxl*, implying that BmSxl acts downstream of BmPrmt5 and BmVasa to regulate apyrene sperm development. These findings indicate that BmPrmt5 and BmVasa constitute an integral regulatory module essential for spermatogenesis in *B*. *mori*.

## Author summary

In lepidopteran insects, males produce both nucleated and anucleated sperm. Both sperm morphs are essential for fertilization: The eupyrene sperm fertilizes the egg, and the apyrene sperm assist in transport of the eupyrene sperm to the female sperm-storage organs. In this study, we defined the function of the arginine methyltransferase Prmt5 and its substrate Vasa in the lepidopteran insect *Bombyx mori*. Our genetic analyses revealed that both *BmPrmt5* and *BmVasa* are essential for spermatogenesis. Combined with RNA-seq, genetic, immunofluorescence staining, and mass spectrometry analyses suggest that the BmPrmt5–BmVasa module regulates spermatogenesis in *B*. *mori*. This study expands our knowledge of reproduction biology in insects and identifies potential gene targets for pest control.

## Introduction

Spermatogenesis is a vital process for sexually reproducing animals [1]. During spermatogenesis, the germ cells undergo mitosis and meiosis and complete morphological changes resulting in mature sperm [2,3]. Despite the central role of spermatozoa in reproduction, sperm cells exhibit exceptional diversity across species at both ultrastructure and molecular levels, making a valuable model for molecular genetics research [4,5]. Yet most studies on the mechanism of spermatogenesis have focused on model species such as mice, zebrafish, and Drosophila [1,6], which limits our knowledge concerning the developmental genetics underlying the evolutionary diversity of sperm.

In pursuing novel insights into the diversity of spermatogenesis, Lepidoptera (moths and butterflies) is an emerging and notable taxon. Moreover, understanding reproductive processes in Lepidoptera is valuable because it includes both pest species and species of economic importance [7,8]. Lepidotperan males exhibit dichotomous spermatogenesis characterized by the production of eupyrene (nucleate) and apyrene (anucleate) spermatozoa [9]. Both sperm morphs are essential for fertilization: the eupyrene sperm fertilize the egg, and the apyrene sperm is necessary for the transport of eupyrene sperm to the female sperm-storage organs [10,11]. Such sperm dimorphism is thought to be shared by nearly all lepidopteran species [12], except for the most early-diverging lineages [13]. Like most other lepidopteran species, the silkworm moth *Bombyx mori* (*B*. *mori*) displays dichotomous spermatogenesis. To date, the studies on dichotomous spermatogenesis in *B*. *mori* and other lepidopteran species have been limited mostly to aspects related to cytology and developmental timing carried out by microscopic observations. Only few genes such as *poly(A)-specific ribonuclease-like domain-containing 1* (*BmPnldc1*), *Hua enhancer 1* (*BmHen1*), *Sex-lethal* (*BmSxl*), *Maelstrom* (*BmMael*), and *Polyamine modulated factor 1 binding protein* (*BmPMFBP1*) have been experimentally linked to this process in *B*. *mori* [10,11,14–20]. Therefore, the molecular and genetic framework that controls spermatogenesis remains largely unknown, and it is important to continue investigating additional genes and molecular mechanisms that may be governing dichotomous spermatogenesis.

Post-translational modifications play critical roles in diverse cellular events including the DNA damage response, chromosome condensation, and cytoskeletal organization during germ cell differentiation [21,22]. The type II arginine methyltransferase Protein arginine methyltransferase 5 (Prmt5) catalyzes the symmetrical dimethylation of arginine residues (sDMA) on its protein substrates [23,24]. Methylated arginines, in particular sDMAs, mediate the binding of Tudor domains of proteins to regulate protein-protein interactions and protein cellular localization [25–27]. As a catalyst of sDMA installation, Prmt5 has been shown to play important roles in developmental processes in a number of species [24,28,29]. In mouse, it is required for early embryonic development and stem cell differentiation [30–32]. In *Drosophila melanogaster*, mutation of *Dart5*, a *Prmt5* homolog, results in grandchild-less females and male spermatogenesis defects, as well as disruption of the circadian rhythms in locomotor activity [33–36]. In zebrafish, loss of function of *Prmt5* causes a reduction in germ cell number, leading to the failure of gonads to differentiate into normal testis or ovaries and eventual sterility [37]. The function of Prmt5 is previously unexplored in *B*. *mori* or other insects other than *Drosophila*. Given the precedent that Prmt5 plays a key role in development and gametogenesis in such diverse species, we hypothesize a similar role in *B*. *mori*, and in particular seek to assess if it regulates aspects of dichotomous spermatogenesis.

A number of Prmt5 substrates have been identified, through biochemical and genetic studies, to function in regulating germ cell specification [21]. Notable examples include Vasa, P-element induced wimpy testis (PIWI) proteins, and Tudors-domain-containing proteins from species as diverse as in *Drosophila*, *Xenopus*, and mouse [26,38]. Of particular note, Vasa is a widely-conserved member of the DEAD-box family protein that functions as an ATP-dependent RNA helicase [39]; it is specifically expressed in the germline of *Drosophila* and is required for the assembly and function of the pole plasm during oogenesis [40–43]. The *Xenopus* Vasa homolog is expressed in oocytes and embryos and is required for the formation of germ cells [44–46]. The expression of MVH (also known as DDX4), the mouse homolog of Vasa, is also restricted to the germ cell lineage, and loss of MVH function causes a deficiency in the proliferation and differentiation of spermatocytes [47]. Like Prmt5, whether Vasa functions in spermatogenesis in insects other than *Drosophila* is unknown, and it is of particular interest to assess its role in the dichotomous spermatogenesis of Lepidoptera.

In this study, we characterized the functions of BmPrmt5 and BmVasa in the regulation of spermatogenesis in *B*. *mori*. We first generated loss-of-function mutants of *BmPrmt5* and *BmVasa* through CRISPR/Cas9-based gene editing. The male *BmPrmt5* mutants have severe defects in spermatogenesis, and both males and females are sterile. We showed by immunofluorescence staining that BmVasa is expressed in sperm cysts throughout spermatogenesis. The *BmVasa* mutants also have severe defects in spermatogenesis, and both sexes are sterile. To gain insights into the changes in global gene expression associated with spermatogenesis defects in *BmPrmt5* and *BmVasa* mutants, we performed RNA-seq analysis and found that levels of mRNAs encoded by numerous cell-differentiation-related genes are significantly decreased. The combined results of our genetic, immunofluorescence staining, and RNA-seq analyses of *BmPrmt5* and *BmVasa* mutants demonstrate that the observed defects in eupyrene and apyrene sperm result from disorganized cellular structural proteins and cell polarity disorders. Thus, in *B*. *mori* and likely other lepidopterans, BmPrmt5–BmVasa regulatory module is essential for spermatogenesis.

## Results

### Mutation of *BmPrmt5* leads to both female and male sterility in *B*. *mori*

**Fig 1:**
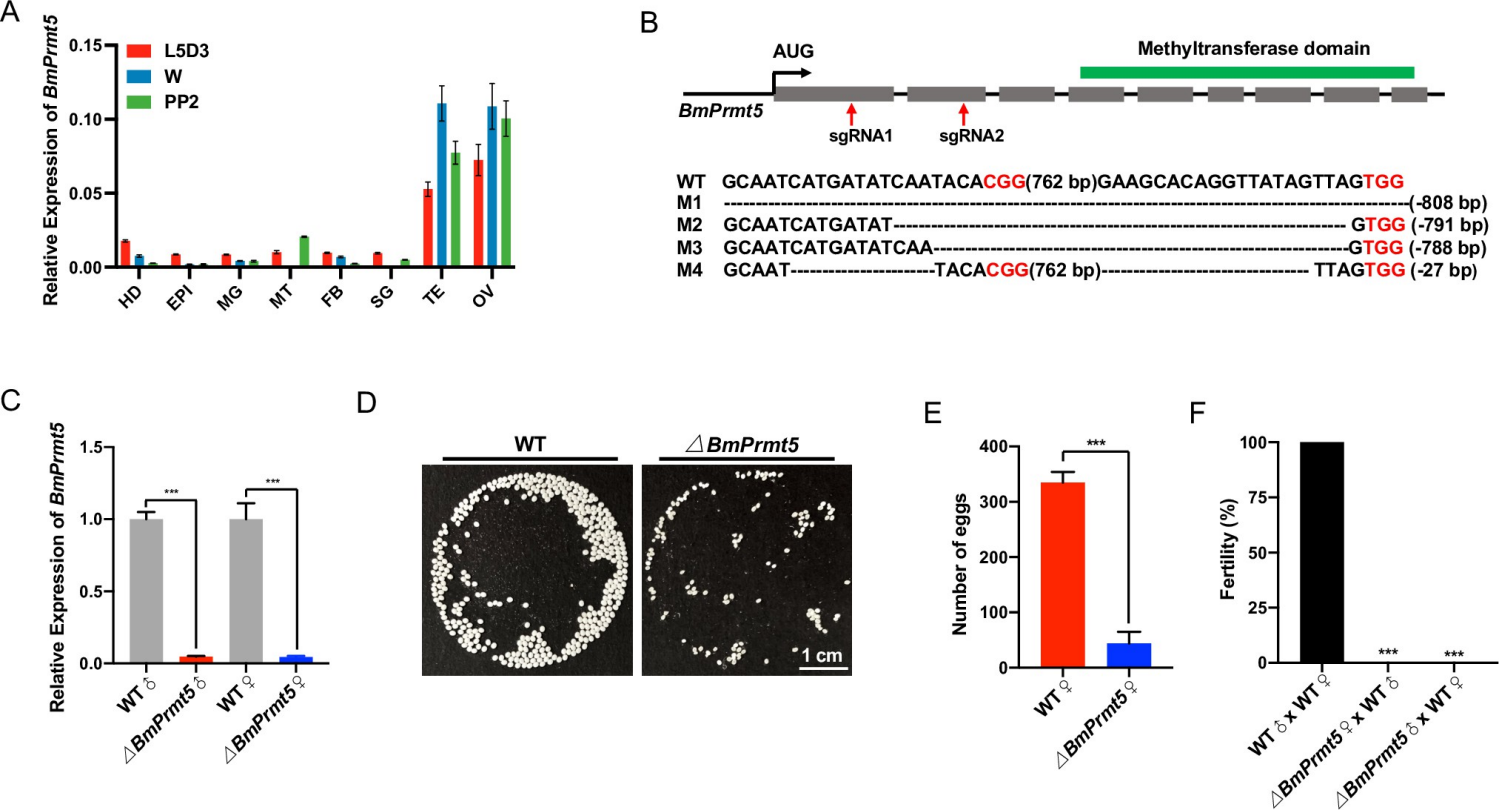
Mutation of *BmPrmt5* causes both male and female sterility in *B*. *mori*. (A) qRT-PCR analysis of *BmPrmt5* mRNA levels in eight tissues at three different stages. *BmRp49* was analyzed as an internal reference. Data are means ± SEM. Abbreviations: HD, head; EPI, epidermis; MG, midgut; MT, malpighian tubule; FB, fat body; SG, silk gland; TE, testis; OV, ovary; L5D3, the third day of the fifth larval stage; W, wandering stage; PP2, the second day of the prepupal stage. (B) Upper: Schematic of the *BmPrmt5* gene. Gray-filed boxes represent exons, lines represent introns. Location of sequence encoding methyltransferase domain predicted by SMART is highlighted with a green box above the gene structure. Red arrows indicate the target sites of sgRNA1 and sgRNA2. Lower: Sequences of *BmPrmt5* gene from WT and four mutant lines. The dashed lines indicate deleted sequences. The PAM sequence is shown in red. (C) qRT-PCR analysis of *BmPrmt5* mRNA in testis and ovary of WT and *BmPrmt5* mutant wandering stage individuals. Error bars are means ± SEM (n = 3, *P* < 0.001, t-test). (D) Photographs of eggs laid by WT and *BmPrmt5* mutant females mated with WT males. The eggs laid by *BmPrmt5* mutant females did not develop. Scale bar, 1 cm. (E) The number of eggs laid by WT and *BmPrmt5* mutant females mated with WT males (n = 6, *P* < 0.001, t-test). (F) Fertility of males and females of the indicated genotypes (n = 15, *P* < 0.001, Fisher’s exact test). *ΔBmPrmt5* represents *BmPrmt5* mutants.

A previous study showed that Prmt5 is predominantly expressed in the germline of *Drosophila* [33]. To evaluate whether *BmPrmt5* has an organ-specific expression pattern in *B*. *mori*, we performed qRT-PCR analysis of samples prepared from different organs. *BmPrmt5* was predominantly expressed in the gonads at all developmental stages analyzed (Fig 1A). To evaluate *BmPrmt5* function, we used a binary transgenic CRISPR/Cas9 system to obtain the loss-of-function mutant. Two small guide RNAs (sgRNAs) targeting exons 1 and 2 of *BmPrmt5* were designed and synthesized, and *BmPrmt5* mutants were generated through genetic crossing between the U6-sgRNA lines and the nos-Cas9 lines (Fig 1B). The region of the gene targeted by the sgRNAs was analyzed by genomic PCR and DNA sequencing in randomly selected F1 offspring. These data showed that there were mutations that altered the open reading frame of *BmPrmt5* in both male and female *BmPrmt5* mutant individuals (Fig 1B). qRT-PCR analysis showed that barely detectable levels of *BmPrmt5* mRNA in *BmPrmt5* larvae mutants (Fig 1C). The *BmPrmt5* mutant adults were viable and no gross abnormalities were observed; however, the *BmPrmt5* mutant females laid significantly fewer eggs than the wild-type (WT) females (Fig 1D and 1E). Both female and male offspring resulting from crosses between *BmPrmt5* mutant females and WT males and between *BmPrmt5* mutant males and WT females were sterile (Fig 1F). These results demonstrate that BmPrmt5 is essential for both female and male fertility.

### BmPrmt5 is essential for spermatogenesis

The demonstration that *BmPrmt5* mutant males are sterile indicates possible defects in the reproduction system. Therefore, we first investigated whether the *BmPrmt5* mutant males exhibited any gross defects in the genitalia or the reproductive system. However, no obvious defects were detected, so we searched for anomalies in spermatogenesis. Sperm of both types develop in distinct cysts containing cells that develop in a coordinated way, eventually elongating into bundles of mature sperm; all sperm in a single bundle develop into the same morph type. Eupyrene and apyrene sperm of *B*. *mori* have different morphologies and also different timings of differentiation during spermatogenesis [15–17]. Specifically, eupyrene spermatogenesis begins on the first day of the fifth instar larval stage and is largely completed by pupation, whereas apyrene spermatogenesis starts during the wandering stage and continues into adulthood. Other than this difference in developmental timing, spermatogenesis appears similar between morphs up until meiosis in spermatocytes [48]. Eupyrene spermatogenesis continues in a manner similar to other insects, producing “typical” sperm with DNA condensed in the nucleus at the sperm head. However, apyrene sperm show major meiotic anomalies, after which several “micronuclei” form in each cell, which migrate towards the sperm tail and are eventually extruded along with excess cytoplasm during spermiogenesis.

**Fig 2:**
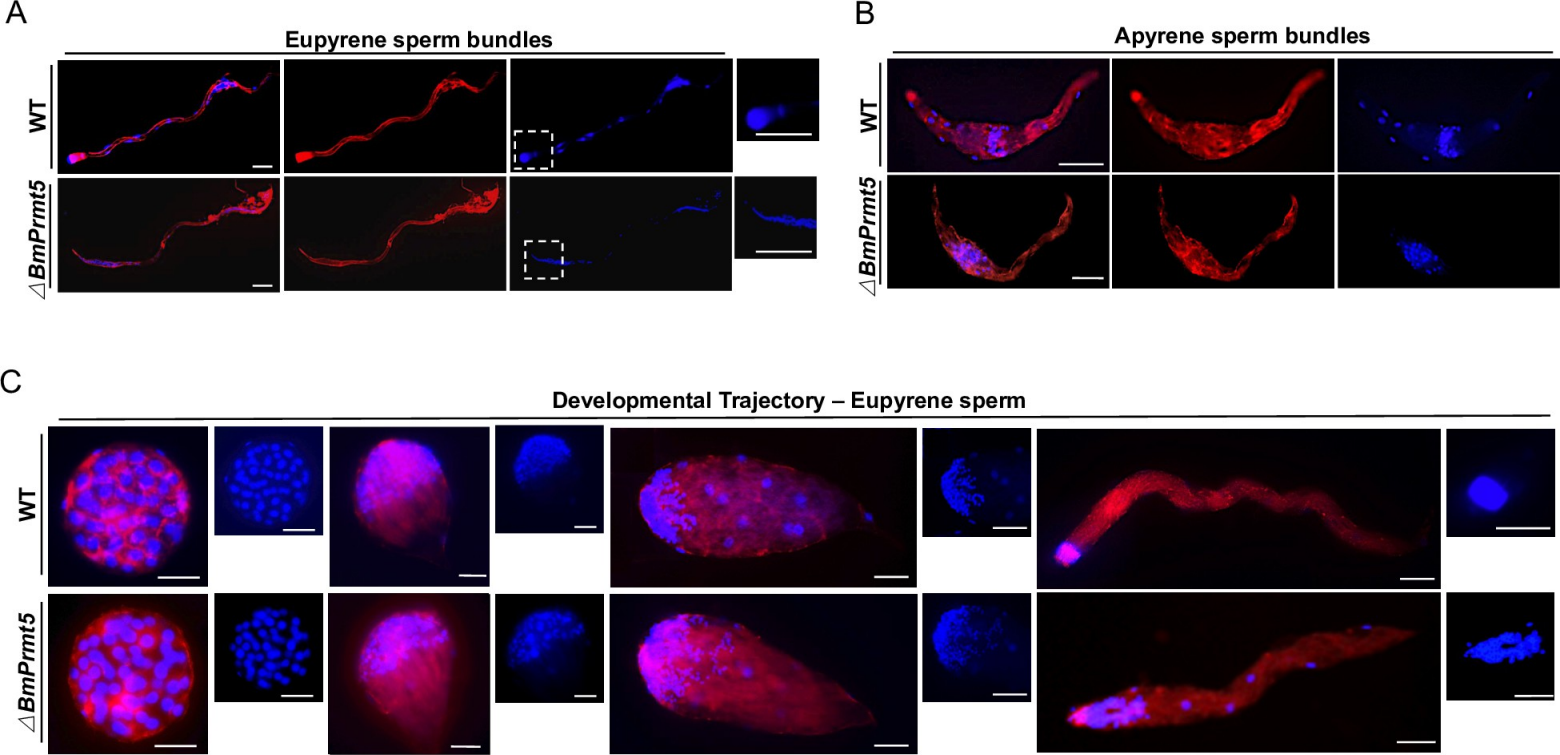
Loss-of-function of *BmPrmt5* leads to defects in spermatogenesis. (A and B) Representative immunofluorescence images of A) eupyrene sperm bundles and B) apyrene sperm bundles from WT and *BmPrmt5* mutants on the seventh day of the pupal stage. Scale bars, 50 μm. Eupyrene sperm head are highlighted in dash boxes. (C) Representative immunofluorescence images of the development trajectory of eupyrene sperm bundles from WT and *BmPrmt5* mutants at day four of the fifth larval instar. Blue, Hoechst; Red, F-actin. Scale bars, 50 μm. *ΔBmPrmt5* represents *BmPrmt5* mutants.

To determine whether *BmPrmt5* is required for spermatogenesis, we first performed fluorescence staining to examine the development of both eupyrene and apyrene sperm bundles on the seventh day of the pupal stage in WT and *BmPrmt5* mutant males. The results showed that the sperm nuclei were assembled regularly at the head of the eupyrene sperm bundles in the WT, whereas in *BmPrmt5* mutants the eupyrene sperm nuclei were abnormally organized and exhibited squeezed eupyrene sperm bundles (Fig 2A). The apyrene sperm of WT had no polarity, and small round micronuclei were distributed in the middle region (Fig 2B). By contrast, most of the apyrene sperm bundles in *BmPrmt5* mutants had defects in sperm nucleus shape and localization (Fig 2B), and only a few normal apyrene sperm were observed in the testis at the late pupae stage.

Next, we investigated eupyrene spermatogenesis in the testes on the fourth day of the fifth larval instar in WT and *BmPrmt5* mutants in detail. At the early elongating stage, *BmPrmt5* mutant eupyrene sperm bundles were similar to those from WT males with round nuclei localized in the anterior region of the bundles (Fig 2C). At the late elongating stages, however, the *BmPrmt5* mutant eupyrene sperm nuclei were abnormally located throughout the bundle (Fig 2C). Taken together, these results demonstrate that *BmPrmt5* is critical for spermatogenesis.

### BmVasa is expressed in spermatocytes and sperm bundles throughout spermatogenesis and is necessary for reproduction in *B*. *mori*

**Fig 3:**
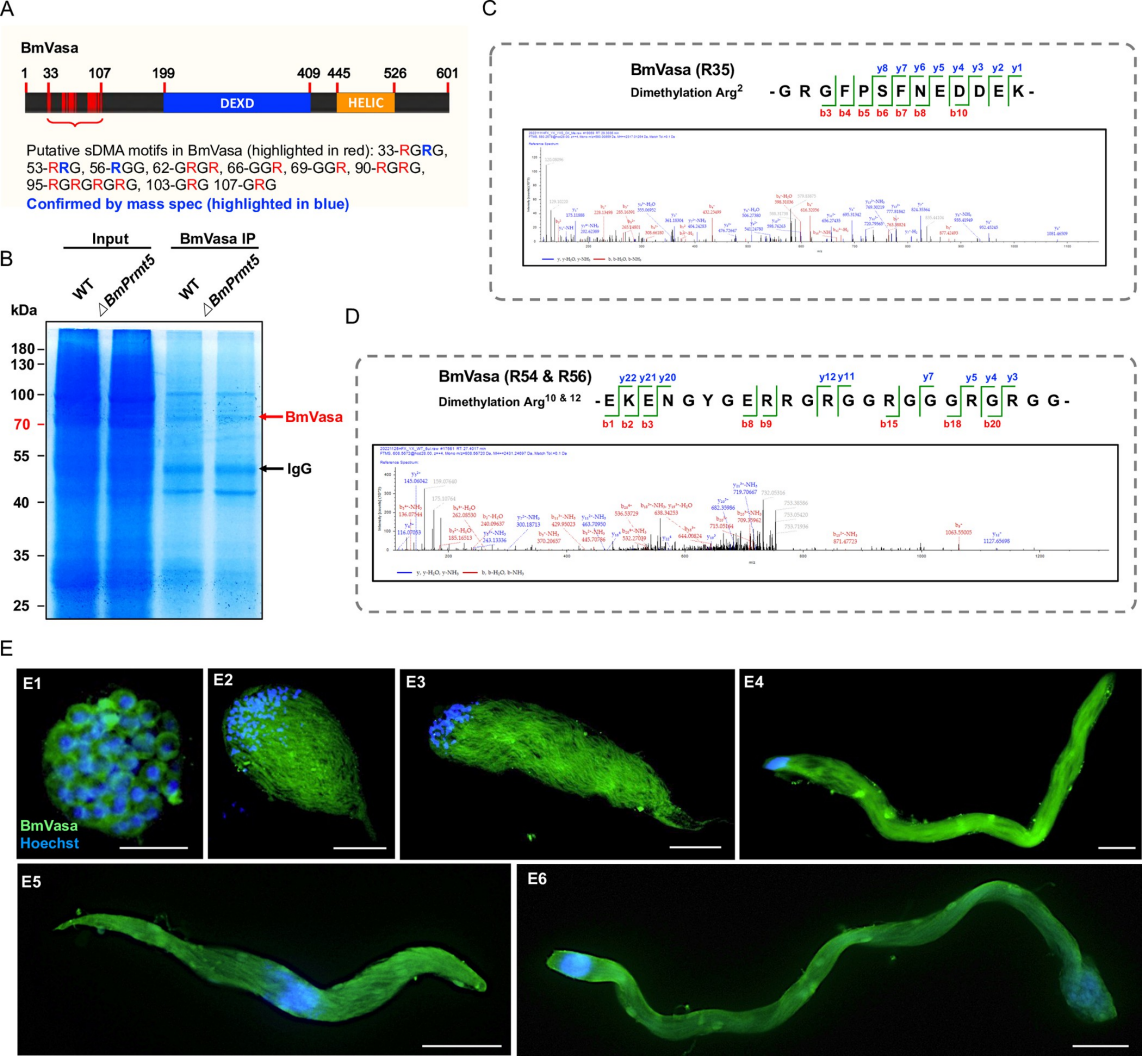
*BmVasa* is expressed in spermatocytes and sperm bundles throughout spermatogenesis in *B*. *mori*. (A) Schematic of BmVasa and putative sDMA sites. Numbers refer to amino acid positions. DEXD, DEAD-like helicase superfamily domain; HELIC, helicase superfamily C-terminal domain. The verified dimethylated site (R35, R54, and R56) is highlighted in blue. (B) SDS-PAGE gel image of samples after endogenous BmVasa immunoprecipitation. Testis-extracted proteins from WT and *BmPrmt5* mutant animals at the fourth day of the fifth larval stage were used for immunoprecipitation with BmVasa antibody. *ΔBmPrmt5* represents *BmPrmt5* mutants. (C and D) Corresponding MS/MS spectra of dimethylated peptides of BmVasa annotated with a comprehensive series of b and y fragment ions. (E) Representative immunofluorescence images of spermatocytes and sperm bundles. E1. spermatocytes on the fourth day of the fifth instar larval stage; E2-E4, elongating eupyrene sperm bundles on the fourth day of the fifth instar larval stage; E5, apyrene sperm bundles on the seventh day of pupal stage; and E6, eupyrene sperm bundles on the seventh day of pupal stage. Blue, Hoechst; Green, anti-BmVasa. Scale bars, 50 μm.

In diverse animal species, Prmt5 catalyzes dimethylation of substrates, including Vasa, to regulate their activity in gonad tissues [35–37,49]. It is also reported that this process involves a physical interaction between BmPrmt5 and BmVasa proteins [50]. We first analyzed the putative sDMA motifs in BmVasa by aligning Vasa homologs in lepidopterans and other species in which Prmt5 activity has been characterized including *Caenorhabditis elegans*, *Danio rerio*, *Xenopus laevis*, *Mus musculus*, and *D*. *melanogaster* (S1 Fig). The results demonstrate that there is a conserved sDMA motif in the N-terminal region of BmVasa (Fig 3A). To further identify the specific dimethylated arginine residues of BmVasa induced by BmPrmt5, we performed mass spectrometry analysis of BmVasa after co-immunoprecipitation using anti-BmVasa antibody in WT and *BmPrmt5* mutant testis extracted protein (Fig 3B). We found multiple arginine residues, including R35, R54, and R56 of BmVasa were dimethylated in WT while unmethylated in *BmPrmt5* mutants (Figs 3C and 3D and S2).

To explore whether the Prmt5 and Vasa might act as a module to regulate spermatogenesis in the *B*. *mori*, we analyzed the expression patterns of *BmPrmt5* and *BmVasa*. qRT-PCR analysis of samples prepared from different organs showed that *BmVasa* was predominantly expressed in the gonads at all stages analyzed (S3A and S3B Fig). In testis at the different stages during the spermatogenesis, levels of both *BmPrmt5* and *BmVasa* mRNAs started to increase on the fifth day at the fifth larval stage and peaked at the wandering stage, and then gradually decreased (S3A and S3B Fig). We then used an antibody against Vasa to detect the cellular localization of BmVasa in spermatocytes by immunohistochemistry; the protein was localized in the cytoplasm and was expressed in sperm cysts throughout different development stages (Figs 3E and S4).

**Fig 4:**
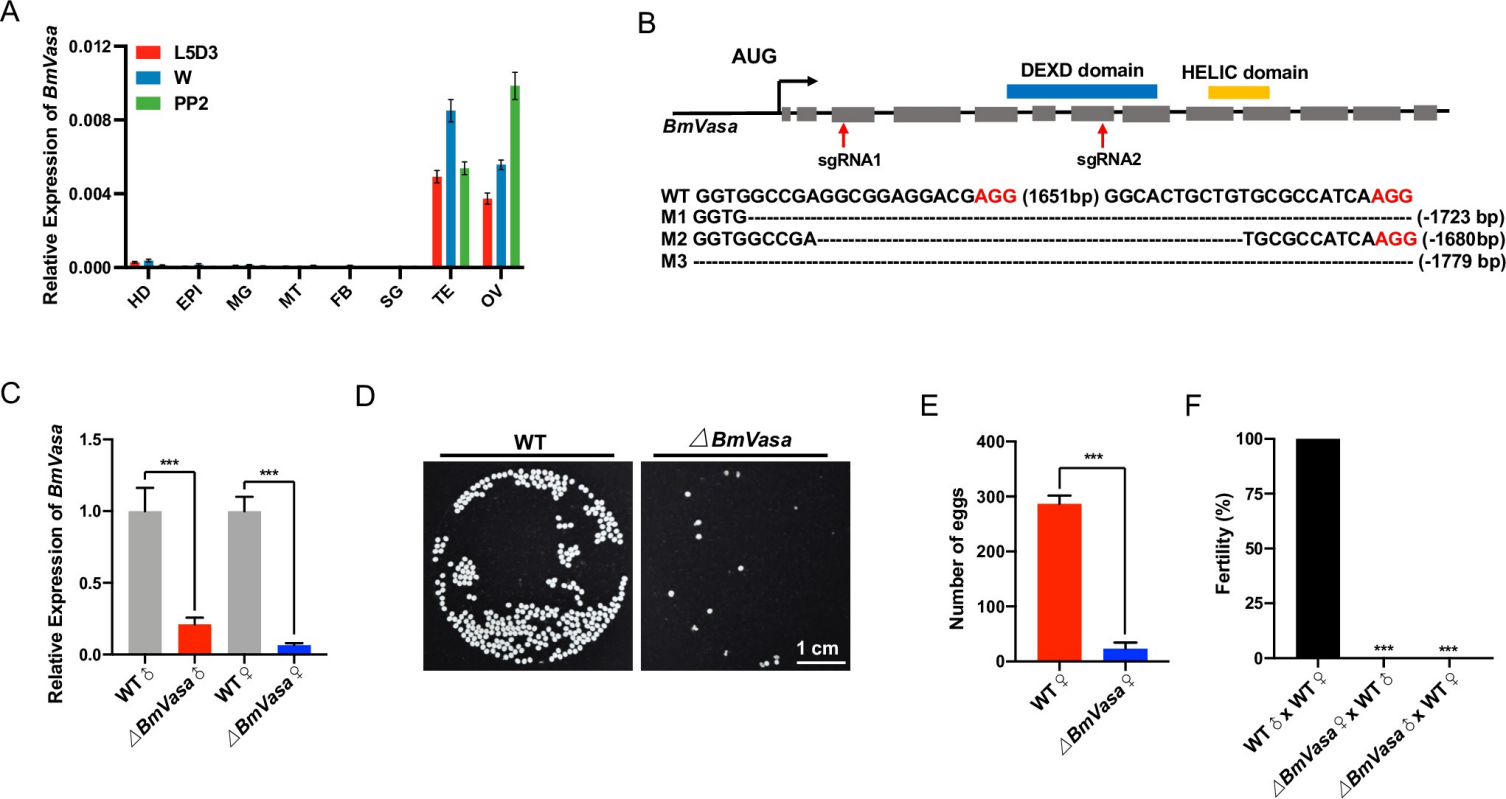
Loss-of-function of *BmVasa* causes both male and female sterility in *B*. *mori*. (A) qRT-PCR analysis of *BmVasa* mRNA levels in eight tissues at three different stages. HD, head; EPI, epidermis; MG, midgut; MT, malpighian tubule; FB, fat body; SG, silk gland; TE, testis; OV, ovary; L5D3, the third day of the fifth larval stage; W, wandering stage; PP2, the second day of the prepupal stage. *BmRp49* was used as an internal reference. Data are means ± SEM. (B) Upper: Schematic of *BmVasa* gene structure. Gray-filed boxes represent exons, and lines represent introns. DEXD and HELIC domains predicted by SMART are highlighted with blue and yellow boxes above the gene schematic. Red arrows indicate the target sites of sgRNA1 and sgRNA2. Lower: Sequences of *BmVasa* gene from WT and four mutant lines. The dashed lines indicate deleted sequences. The PAM sequence is shown in red. (C) qRT-PCR analysis of *BmVasa* mRNA in testis and ovary of WT and *BmVasa* mutant wandering stage individuals. Error bars are means ± SEM (n = 3, *P* < 0.001, t-test). (D) Images of eggs laid by WT and *BmVasa* mutants. The eggs laid by *BmVasa* mutant females did not develop. (E) The number of eggs laid by WT and *BmVasa* females bred to WT males (n = 6, *P* < 0.001, t-test). (F) Fertility of males and females of the indicated genotypes (n = 15, *P* < 0.001, Fisher’s exact test). *ΔBmVasa* represents *BmVasa* mutants.

To further interrogate the role of BmVasa, we created loss-of-function mutants of *BmVasa* using the CRISPR/Cas9 system. Two sgRNAs targeting exons 3 and 7 of *BmVasa* were designed, and *BmVasa* mutants were generated by crossing U6-sgRNA lines and the nos-Cas9 lines (Fig 4B). Mutations in randomly selected representative F1 offspring were characterized by genomic PCR and sequencing using gene-specific primers (Fig 4B), and qRT-PCR and western blotting analyses showed that the *BmVasa* transcript and the BmVasa protein were barely detectable in *BmVasa* mutants (Figs 4C and S5). The *BmVasa* mutant adults were viable and grossly normal; however, and the *BmVasa* mutant females laid significantly fewer eggs than WT (Fig 4D and 4E). Fecundity tests revealed that both *BmVasa* mutant females and *BmVasa* mutant males were sterile (Fig 4F). These results demonstrate that, like *BmPrmt5*, *BmVasa* is essential for both female and male fertility.

### BmVasa is required for spermatogenesis

**Fig 5:**
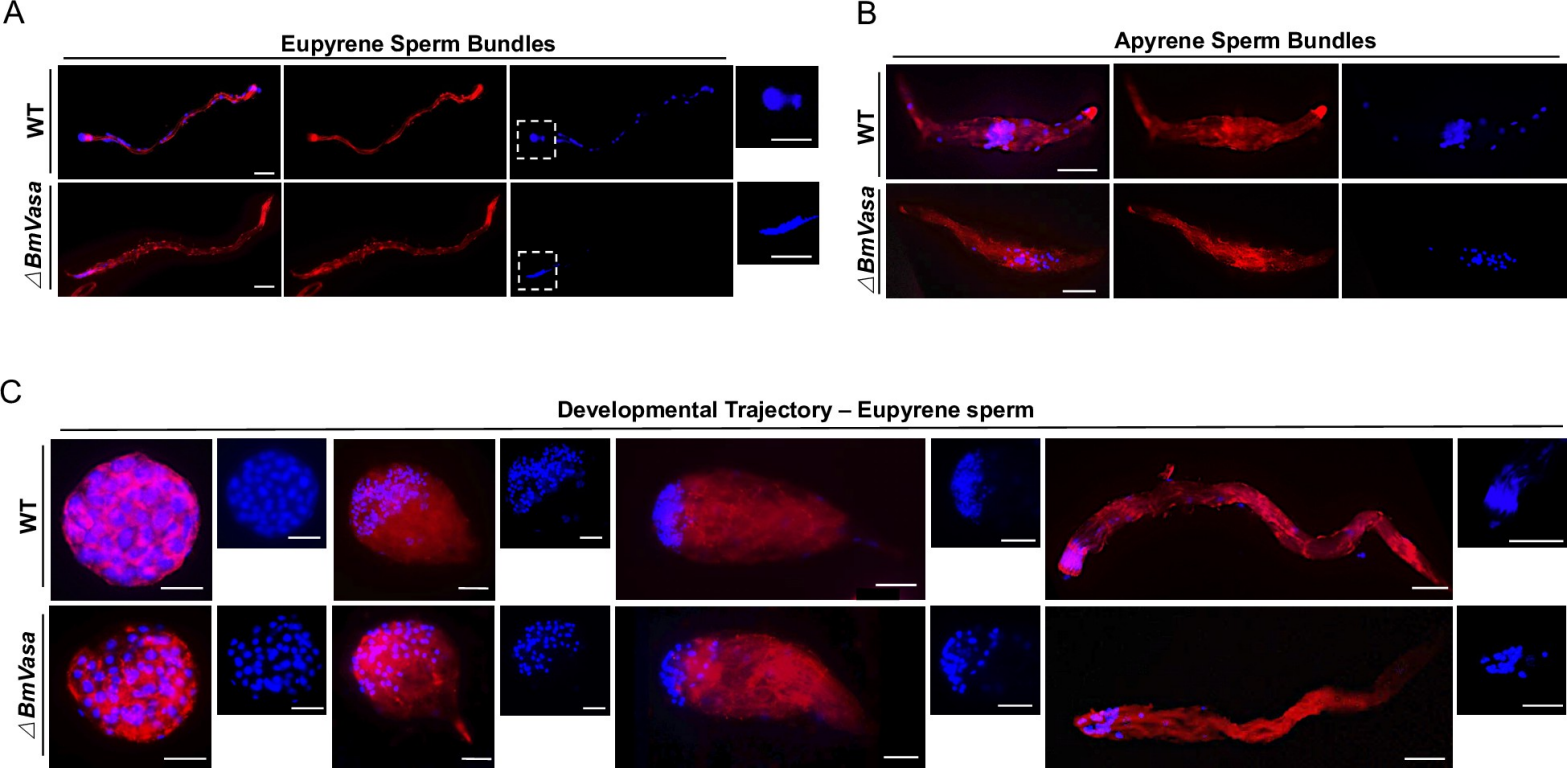
*BmVasa* mutation results in spermatogenesis defects. (A and B) Representative immunofluorescence images of A) eupyrene sperm bundles and B) apyrene sperm bundles from WT and *BmVasa* mutants on the seventh day of the pupal stage. Scale bars, 50 μm. Eupyrene sperm head are highlighted in dash boxes. (C) Representative immunofluorescence images of the development trajectory of eupyrene sperm bundles at different developmental stages from WT and *BmVasa* mutants at day four of the fifth larval instar. Blue, Hoechst; Red, F-actin. Scale bars, 50 μm. *ΔBmVasa* represents *BmVasa* mutants.

To further explore how *BmVasa* influences spermatogenesis, we examined the development of both eupyrene and apyrene sperm bundles on day seven of the pupal stage in WT and *BmVasa* mutant males by fluorescence staining. Both eupyrene and apyrene sperm bundles in *BmVasa* mutants showed severe abnormalities. Like *BmPrmt5* mutant eupyrene sperm, those from *BmVasa* mutants had abnormally organized sperm nuclei (Fig 5A). Moreover, like *BmPrmt5* mutants, all the *BmVasa* mutant apyrene sperm bundles had severe defects in shape and location of micronuclei, and some of the defective sperm bundles displayed intermediate morphology between normal apyrene sperm and early elongating eupyrene sperm bundles (Fig 5B).

Next, we investigated eupyrene spermatogenesis in the testis on day four of the fifth larval instar. At the early elongating stage, *BmVasa* mutant eupyrene sperm bundles were similar to those of WT, with round nuclei localized in the anterior part of the bundles, but in the late elongating stages, the nuclei in *BmVasa* mutants were abnormally localized (Fig 5C). Thus, mutation of *BmVasa* and mutation of *BmPrmt5* had very similar effects on spermatogenesis.

### BmPrmt5 does not affect the transcript and protein level of BmVasa

To determine whether BmPrmt5 regulates the expression of *BmVasa*, we performed qRT-PCR analysis of *BmPrmt5* mutant testis and sperm for *BmVasa* mRNA. *BmVasa* expression was similar in WT and *BmPrmt5* mutant larvae (S6A and S6B Fig). We then determined the levels of BmVasa protein in WT and *BmPrmt5* mutant larvae by western blotting using an anti-BmVasa antibody. BmVasa was expressed at a similar level in these two backgrounds (S6C Fig). These results indicate that BmPrmt5 does not influence the production or stability of BmVasa.

### BmPrmt5 and BmVasa co-regulate a large number of genes

**Fig 6:**
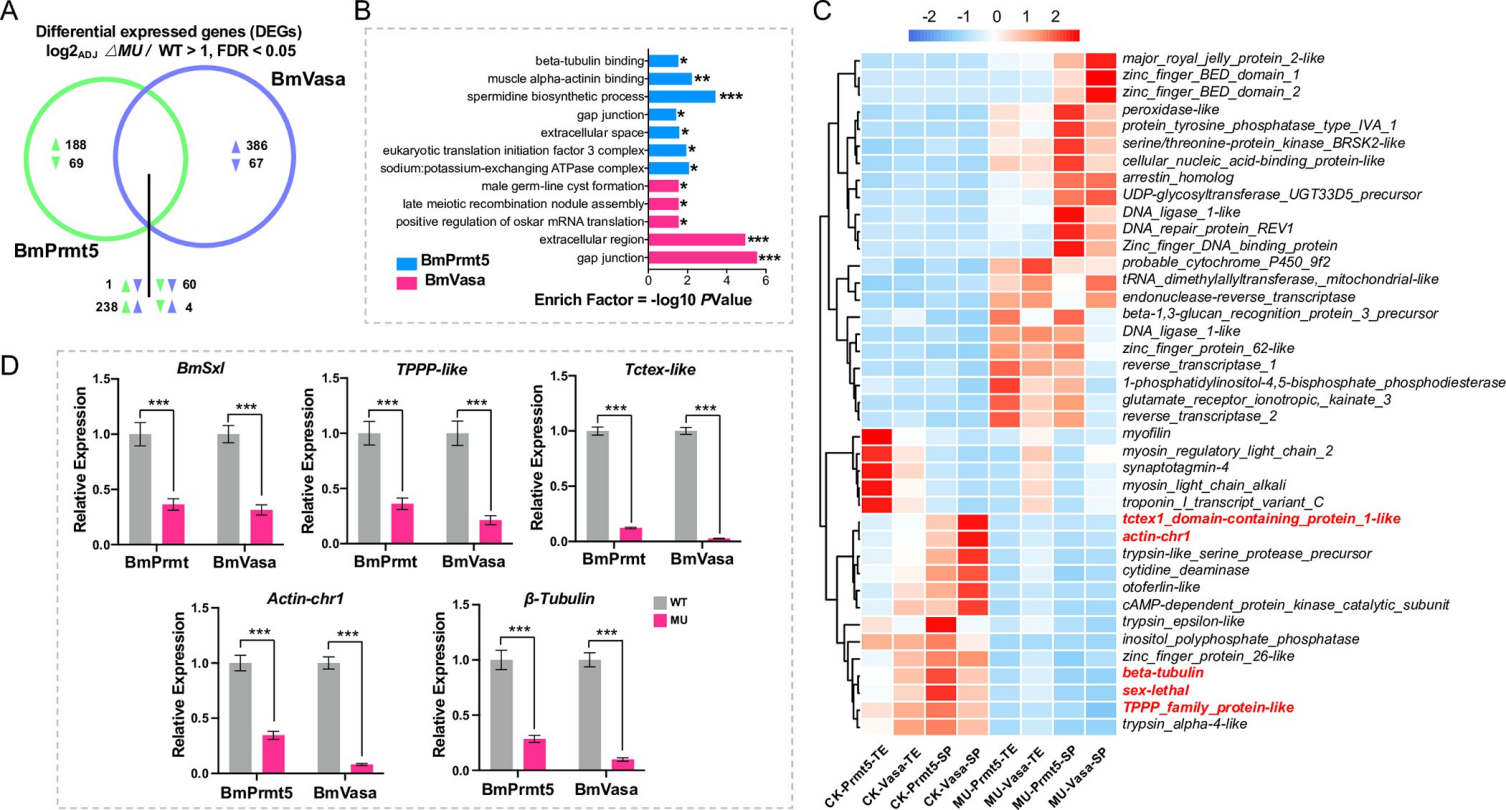
BmPrmt5 and BmVasa co-regulate many spermatogenesis-related genes. (A) Venn diagram of numbers of sperm-specific genes commonly and specifically regulated by BmPrmt5 (left, green) and BmVasa (right, purple) relative to WT. (B) GO enrichment analysis of the biological processes associated with the sperm-specific genes regulated by BmPrmt5 and BmVasa. * *P* ≤ 0.05; ** *P* ≤ 0.01; *** *P* ≤ 0.001. (C) Hierarchical clustering of selected spermatogenesis-related DEGs in different samples. CK-Prmt5, control for *BmPrmt5* mutants; CK-Vasa, control for *BmVasa* mutants; MU-Prmt5, *BmPrmt5* mutants; MU-Vasa, *BmVasa* mutants; TE, testis; SP, sperm. (D) qRT-PCR analysis of the selected spermatogenesis-related DEGs regulated by BmPrmt5 and BmVasa. Data are means ± SEM (n = 3, *** *P* < 0.001, two-tailed Student’s t-test). *ΔBmPrmt5* and *ΔBmVasa* represent *BmPrmt5* and *BmVasa* mutants, respectively.

To gain insights into the changes in global gene expression associated with mutations of *BmPrmt5* and *BmVasa*, we performed RNA-seq assays of spermatocytes/sperm bundles isolated from testis of WT, *BmPrmt5*, and *BmVasa* mutants. In *BmPrmt5* and *BmVasa* mutants, after removing background, 404 and 522 ‘sperm-specific’ genes that were differentially expressed compared to WT were identified, respectively (S7 Fig). Among these differentially expressed genes (DEGs), 303 were co-regulated by BmPrmt5 and BmVasa; of these 238 (78%) were up-regulated and 60 (19%) were down-regulated (Fig 6A). Next, we performed gene ontology (GO) enrichment analysis and found that the genes regulated by both BmPrmt5 and BmVasa were preferentially associated with gap junctions, components of the extracellular region, and germ-line cyst formation (Fig 6B). After the unannotated and transposon-related DEGs co-regulated by BmPrmt5 and BmVasa were removed, hierarchical clustering analyses of the remaining genes revealed that 35 of these genes were regulated by BmPrmt5 and BmVasa in the same direction in both testis and sperm (Fig 6C). Among the genes significantly down-regulated by BmPrmt5 and BmVasa, some are related to spermatogenesis, including those encoding BmSxl, TPPP-like, Tctex-like, Actin-chr1, and β-Tubulin. BmSxl is an RNA-binding protein, whose mutation leads to severe defects in apyrene sperm development in *B*. *mori* [10,11]. The TPPP-like protein, TPPP2, is crucial for sperm morphogenesis in mice [51]. In *Drosophila*, the ortholog of the Tctex-1 acts as the dynein light chain, and its null mutant displays a diffused nuclear sperm cyst [52,53]. We verified the expression of *BmSxl*, *TPPP-like*, *Tctex-like*, *Actin-chr1*, and *β-Tubulin* using qRT-PCR from sperm bundles isolated from WT, *BmPrmt5*, and *BmVasa* mutants at the fourth day of the fifth instar. Expression of all these genes was severely repressed in *BmPrmt5* and *BmVasa* mutants (Fig 6D), confirming that both BmPrmt5 and BmVasa promote the expression of these genes.

**Fig 7:**
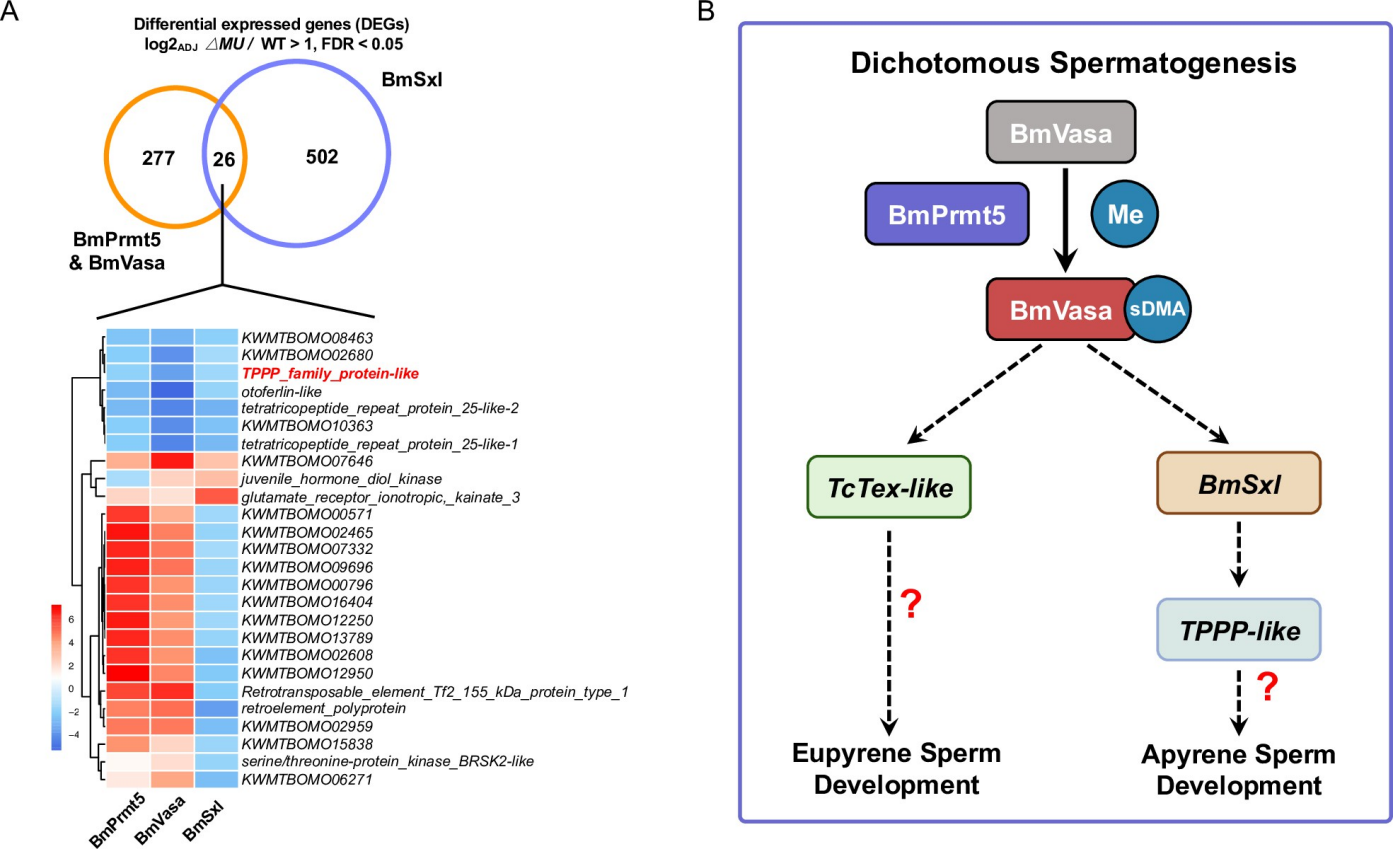
Proposed model for the functions of the BmPrmt5–BmVasa module in *B*. *mori*. (A) Upper: Venn diagram of numbers of sperm-specific genes commonly and specifically regulated by BmSxl (right, purple) and BmPrmt5–BmVasa module (left, orange) relative to WT. Lower: Hierarchical clustering analysis of genes regulated by BmPrmt5, BmVasa, and BmSxl. The fold change of each gene in different samples is shown in the heatmap (blue, down-regulated; red, up-regulated). (B) Model illustrating how the BmPrmt5–BmVasa module may regulate dichotomous spermatogenesis in *B*. *mori*. BmPrmt5 catalyzes the sDMA of residues in BmVasa. As a result, BmVasa enhances the expression of spermatogenesis-related genes such as *BmSxl*, *BmTPPP-like*, and *BmTctex-like* to promote eupyrene and apyrene sperm development.

As BmSxl plays a critical role in regulating apyrene sperm development in *B*. *mori* [10,11], we asked whether BmSxl might also regulate expression of the genes co-regulated by BmPrmt5 and BmVasa by comparing the 303 DEGs co-regulated by BmPrmt5 and BmVasa with the 528 BmSxl-regulated DEGs identified previously [11]. We found that 26 DEGs were co-regulated by BmPrmt5, BmVasa, and BmSxl (Fig 7A). Hierarchical clustering analyses revealed that nine of these DEGs, including genes encoding TPPP family-like proteins, were co-regulated by BmPrmt5, BmVasa, and BmSxl in the same direction (Fig 7A). These results indicate that BmSxl, BmPrmt5, and BmVasa may function in the same pathway to regulate apyrene spermatogenesis and that the defects in spermatogenesis observed in *BmPrmt5* and *BmVasa* mutants are likely due to the reduced expression of genes required for sperm morphogenesis (Fig 7B).

### Discussion

Prmt5 and Vasa are known as components involved in germ cell development in diverse species [35–37,47], and are thus likely be involved in spermatogenesis in *B*. *mori*. Here we demonstrated that BmPrmt5 and BmVasa are both involved in the regulation of spermatogenesis in the lepidopteran insect *B*. *mori*. We generated the loss-of-function mutants of *BmPrmt5* and *BmVasa* through CRISPR/Cas9-based gene editing. Phenotype analyses of these mutants demonstrated that both BmPrmt5 and BmVasa are required for female and male fertility and for proper development of sperm bundles. Previous work established that Prmt5 catalyzes dimethylation of arginine in PIWI and Tudor proteins in gonads in numerous species across phyla [25,36]. Our mass spectrometry analysis also identified that R35, R54, and R56 of BmVasa were indeed dimethylated in WT while unmethylated in *BmPrmt5* mutants. In *Drosophila* with a mutation in Dart5, the homolog of Prmt5, PIWIs and Tudors are not dimethylated, which compromises their ability to interact with other proteins and to be properly localized in the cells, resulting in severe defects in oogenesis [35,36]. Notably, in our study, we also observed oogenesis defects in *BmPrmt5* and *BmVasa* mutants. Our previous studies have shown that mutation of either PIWIs or Tudors has no effect on male fertility but results in oogenesis defects in *B*. *mori* [20,54,55]. These results indicate that BmPrmt5 may participate in oogenesis via regulating PIWIs and BmVasa. As mutation of BmPrmt5 does not affect the production or stability of BmVasa, we speculate that BmPrmt5-mediated sDMA modification of BmVasa activates BmVasa allowing it to regulate spermatogenesis. We therefore propose that BmPrmt5 and BmVasa act as an integral module to regulate spermatogenesis in *B*. *mori* (Fig 7B).

Previous studies have demonstrated that morphology defects in the bundles of either eupyrene or apyrene sperm compromise their physiological function [10,11,14,19,20]. In *BmPrmt5* and *BmVasa* mutants, there are severe defects in both sperm morphs. RNA-seq analysis indicated that the expression of genes that encode structural proteins are dysregulated in the eupyrene sperm in *BmPrmt5* and *BmVasa* mutants. The phenotypes of *BmPrmt5* and *BmVasa* mutant sperms are similar to those of *LIS-1* mutants and *Tctex-1* mutants in *Drosophila* [52,53]. *BmPrmt5* and *BmVasa* mutant eupyrene sperms also have morphologies similar to those in *BmPnldc1* and *BmHen1* mutants [11,20], although neither BmPnldc1 nor BmHen1 was identified as a DEG in our RNA-seq analysis. The abnormally polarized nuclei we observed in apyrene sperm in *BmPrmt5* and *BmVasa* mutants phenocopy those of *BmSxl* mutants [10,11]. Thus, we propose that the BmPrmt5–BmVasa module regulates spermatogenesis, at least in part, through BmSxl, BmTPPP-like, and BmTctex-like (Fig 7B).

Our study also demonstrated a function of Vasa in Lepidoptera that is not observed in Diptera. In *Drosophila*, Vasa is required for oogenesis and embryo development, but not for male fertility [42,43]. However, our results demonstrate that BmVasa is essential for both female and male fertility in *B*. *mori*. Results from Tribolium, aphids, and bees indicate that Vasa is expressed in gonads [56–58], but these results do not clearly indicate whether our results from *B*. *mori* that the requirement of Vasa for male fertility during spermatogenesis is an exception. Vasa has been proposed to control germ cell formation in *Drosophila* by regulation of the localization and translation of the proteins encoded by pole plasm-related genes via its direct interactions with their RNAs and proteins [58–60]. As apyrene sperm in *BmVasa* mutants have abnormally localized polarized nuclei, some of which show an intermediate phenotype between eupyrene and apyrene sperm bundles, BmVasa may be required for development of the pole plasm of apyrene sperm. We hypothesize that transcription factors mediate the regulation of the expression of *BmSxl* and *BmTctex-like* by the BmPrmt5–BmVasa module, and efforts to identify these postulated transcription factors will be the focus of future studies.

## Materials and methods

### Silkworm strains

The multivoltine, non-diapausing silkworm strain Nistari was used in this study. Larvae were reared on fresh mulberry leaves under standard conditions at 25°C.

### RNA isolation, cDNA synthesis, and qRT-PCR

Total RNA was extracted from three individual mutants or WT animals at the fourth day of the fifth instar using the TRIzol reagent (YEASEN, China) according to the manufacturer’s instructions. An aliquot of 1 μg of the total RNA was used to synthesize cDNA using PrimeScript RT reagent Kit with gDNA eraser (Takara, China). qRT-PCR analysis was performed on a StepOnePlus Real-Time PCR system (Applied Biosystems, USA) with an SYBR green Real-Time PCR master mix (Toyobo, Japan). *Bmrp49* was used as an internal control. The amplification program was as follows: The samples were incubated at 95°C for 5 min, followed by 40 cycles of 95°C for 15 s, and then 60°C for 1 min. Sequences of the qRT-PCR primers are listed in S1 Table.

### Silkworm germline transformation and CRISPR/Cas9-mediated construction of *BmPrmt5* and *BmVasa* mutants

A binary transgenic CRISPR/Cas9 system was used to construct *BmPrmt5* and *BmVasa* mutants. The nos-Cas9 transgenic silkworm lines (nos-Cas9/IE1-EGFP) express the Cas9 nuclease under the control of the *B*. *mori* nanos promoter [61]. The plasmids for expression of two sgRNA (U6-sgRNA/IE1-DsRed) under the control of the *U6* promoter were constructed to generate *BmPrmt5* and *BmVasa* mutants. Primers for plasmid construction and sgRNA targeting sequences are listed in S1 Table.

For silkworm germline transformation, preblastodermal embryos were prepared and microinjected with transgenic plasmids (400 ng/μl) together with helper plasmids (200 ng/μl) and incubated in a humidified chamber at 25°C for 10–12 days until hatching [62,63]. G0 moths were sib-mated or backcrossed with WT moths, and progeny were screened during early larval stages for GFP fluorescence using fluorescence microscopy (Nikon AZ100, Japan).

The nos-Cas9 lines and the U6-sgRNA lines were crossed to generate *BmPrmt5* and *BmVasa* mutants selected based on EGFP and DsRed fluorescence markers. Genomic DNA of the mutated animals was extracted by standard SDS lysis-phenol treatment, incubated with proteinase K, and purified for mutagenesis analysis via PCR amplification with specific primers (S1 Table).

### Phylogenetic and amino acid alignment analysis

Phylogenetic analysis and amino acid alignment analysis were performed using MEGA 7 [64]. For phylogenetic analysis, evolutionary history was inferred using the neighbor-joining method [65]. The percentages of replicate trees in which the associated taxa clustered together in the bootstrap test (1000 replicates) were determined as previously described [66]. The evolutionary distances were computed using the Poisson correction method and are in units of the number of amino acid substitutions per site. Protein sequence of Vasa were downloaded from National Center for Biotechnology Information. The accession numbers are as follows: *Plutella xylostella*, XP_037961717.1; *D*. *rerio*, CAA72735.1; *M*. *musculus*, EDL18409.1; *Tribolium castaneum*, NP_001034520.2; *B*. *mori*, NP_001037347.1; *Manduca sexta*, NP_001037347.1; *X*. *laevis*, NP_001081728.1; *C*. *elegans*, NP_491113.1; *D*. *melanogaster*, NP_723899.1; *Nasonia vitripennis*, XP_001603956.3; *Amyelois transitella*, XP_013187571.1; *Helicoverpa armigera*, XP_021190483.1; *Aedes aegypti*, XP_021700879.1; *Bicyclus anynana*, XP_023939227.1; and *Danaus plexippus*, XP_032519693.1.

### Immunofluorescent staining of sperm bundles

The anti-BmVasa antibody BmVasa-R1 was described previously [55,67]. Immunofluorescence staining experiments using BmVasa-R1 were performed using spermatocytes and sperm bundles isolated from excised testes from the fifth instar larvae stage to adult stage animals. The collected sperm were fixed for 1 h. After two washes with PBS, samples were incubated in the primary antibody-blocking solution mixture overnight at 4°C (PBS containing 0.1% Triton X-100 and 1% bovine serum albumin). After two washes with PBS, samples were incubated with the secondary antibody, TRITC Phalloidin (YEASEN, China), and Hoechst (Beyotime, China) for 1 h at room temperature. Samples were washed three times with PBS and subsequently mounted in the antifade medium (YEASEN, China). Images were taken on an Olympus BX53 microscope (Japan). Antibodies and dilutions used were as follows: BmVasa-R1, 1:200; Alexa Fluor 488 AffiniPure Goat Anti-Rabbit IgG (H+L) (Thermo Fisher, USA), 1:500; TRITC Phalloidin (YEASEN, China), 1:500.

### Western blotting and antibodies

Antibodies and dilutions used were as follows: BmVasa-R1, 1:1000; monoclonal mouse anti-α-tubulin (Thermo Fisher, USA), 1:1000; HRP Goat Anti-Rabbit IgG (H+L) (EpiZyme, China), 1:1000; and HRP Goat Anti-Mouse IgG (H+L) (EpiZyme, China), 1:1000. The Immobilon Western Chemiluminescent HRP substrate Kit (Millipore, USA) was used to detect the protein signal.

### Mass spectrometry analysis

To determine the arginine dimethylation sites of BmVasa, we first conducted co-immunoprecipitation to enrich BmVasa proteins from the testis of WT and *BmPrmt5* mutants at the fourth day of the fifth larval stage (Dynabeads Protein G, Invitrogen, USA). Ten paired testis of WT and *BmPrmt5* mutants were combined as a sample, respectively. We then separated the immunoprecipitates by using 10% SDS-PAGE. After Coomassie Brilliant Blue staining (Beyotime, China), we excised the band corresponding to the BmVasa protein. Purified BmVasa protein was processed by 10 mM TCEP, 50 mM CAA, and then digested by Trypsin or Asp-N at 37°C for 4 h. The concentrated peptides were desalinated and analyzed using Q Exactive HF-X mass spectrometer (Thermo Fisher Scientific, USA). Raw LC-MS/MS data were analyzed using Proteome Discoverer 2.5 (Thermo Fisher Scientific, USA) against BmVasa protein sequences. Two biological replicates of each sample were performed.

### RNA-seq analysis

Total RNA was extracted from the testes and sperm bundles from day four in the fifth larval stage WT, *BmPrmt5* mutant, and *BmVasa* mutant animals. The sperm bundles were released by tearing testis in PBS buffer. RNA was collected from six individual animals from each genotype, and samples from each genotype were pooled. The mRNA was enriched, and then fragmented and used for cDNA synthesis and library construction. The library was sequenced using Illumina Hiseq Sequencing 2000 System. The raw data were qualified, filtered, mapped, and quantified (FastQC, Trimmomatic, Bowtie2, Rsem) to the reference silkworm genome database (http://silkbase.ab.a.u-tokyo.ac.jp/cgi-bin/index.cgi) [68–71], and mRNA abundance was normalized with Deseq2. The calculated gene expression levels were then used to compare gene expression differences between mutant and WT. DEGs were identified based on the Poisson Distribution Method with a false discovery rate (FDR) < 0.05 and the absolute value of log_2_(Y/X) > 1. Enrichment analyses of DEGs were conducted using the gene ontology (GO) analysis [72]. The visualization was processed by using R packages (ggplot2, pheatmap). The RNA-seq raw data were deposited on NCBI SRA database (Accession: PRJNA903799).

### Statistical analysis

All data are expressed as the means ± standard error (SEM). Differences between groups were examined using either a two-tailed Student’s t-test or a two-way analysis of variance. All statistical calculations and graphs were made with GraphPad Prism version 9.

## Supporting information

S1 Table Primers used in this work. (XLSX)

S1 Fig Phylogenetic and amino acid alignment analysis of Vasa proteins from different species. sDMA motifs in aligned sequences are denoted by red arrows.
(TIF)

S2 Fig Extracted ion chromatogram of dimethylated site of BmVasa. The red arrow represents dimethylated sites of BmVasa peptide. *ΔBmPrmt5* represents *BmPrmt5* mutants.
(TIF)

S3 Fig Relative expression pattern of BmPrmt5 and BmVasa in testis. (A and B) qRT-PCR analyses of levels of A) *BmPrmt5* mRNA and B) *BmVasa* in testis from day one of the fifth instar to the third day of the pupal stage.
(TIF)

S4 Fig Representative immunofluorescence staining images of spermatocytes at day four of the fifth larval instar. Green, BmVasa; Blue, Hoechst. Scale bar, 50 μm.
(TIF)

S5 Fig Western blot analysis of BmVasa protein in *BmVasa* mutant. TE and OV denote testis and ovary, respectively. *ΔBmVasa* represents *BmVasa* mutants.
(TIF)

S6 Fig Detection of *BmVasa* transcript and BmVasa protein levels in *BmPrmt5* mutant. (A and B) qRT-PCR analysis of *BmVasa* transcript in *BmPrmt5* mutant. A) testis and B) ovary (C) Western blot analysis of BmVasa protein levels in testis and ovary of *BmPrmt5* mutants. *ΔBmPrmt5* represents *BmPrmt5* mutants.
(TIF)

S7 Fig Overview of DEGs in *BmPrmt5* and *BmVasa* mutant samples. Left: Volcano plots of DEGs in *BmPrmt5* and *BmVasa* mutant testis and sperm samples. Red and blue represent up- and down-regulated DEGs, respectively, with a fold change > 1 and false discovery rate (FDR) < 0.05. Right: Venn diagram of the number of genes commonly and specifically differentially regulated in testis (green) and sperm (blue) samples of *BmPrmt5* mutants and *BmVasa* mutants relative to WT. The “sperm-specific” DEGs were used for further analysis. *ΔBmPrmt5* and *ΔBmVasa* represent *BmPrmt5* and *BmVasa* mutants, respectively.
(TIF)
