# Supplementary figures and images for: The Prmt5-Vasa module is essential for spermatogenesis in Bombyx mori
Source: PLoS Genet. 2023 Jan 12;19(1):e1010600. doi: 10.1371/journal.pgen.1010600 (PMC9876381; doi:10.1371/journal.pgen.1010600)

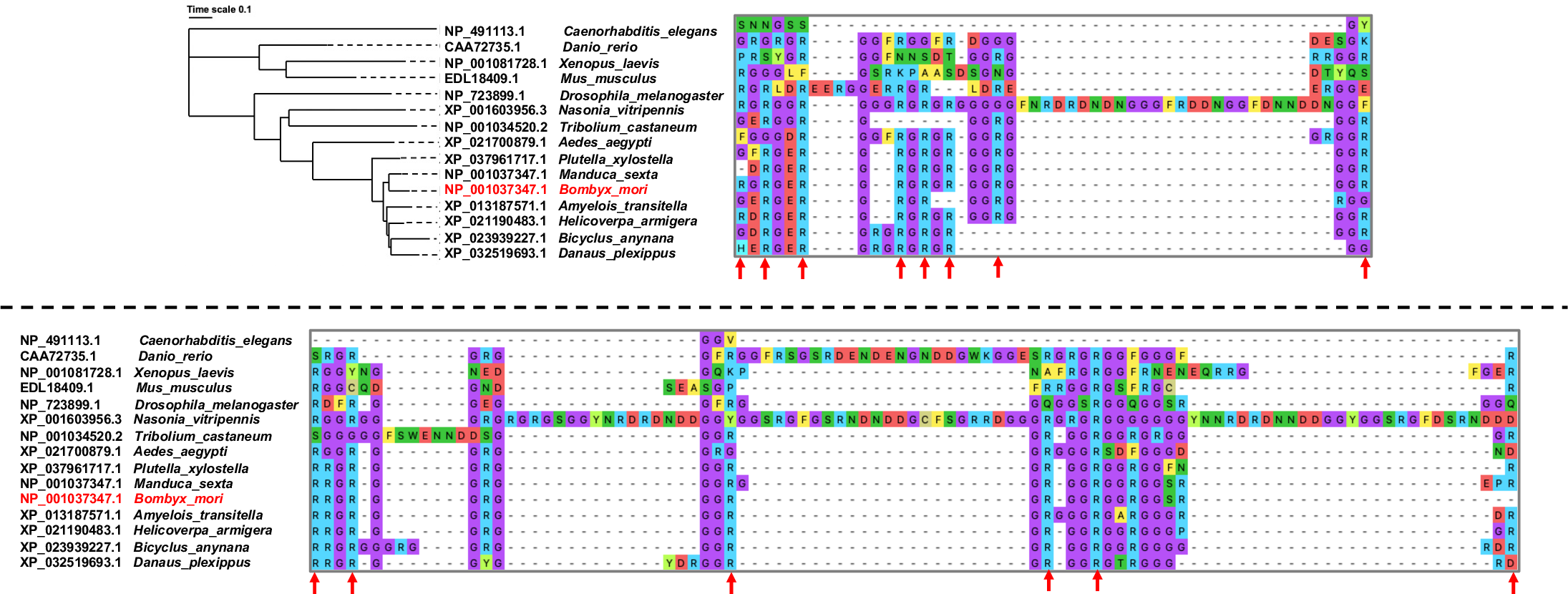

Supplement: S1 Fig — sDMA motifs in aligned sequences are denoted by red arrows. (TIF) [file pgen.1010600.s002.tif]

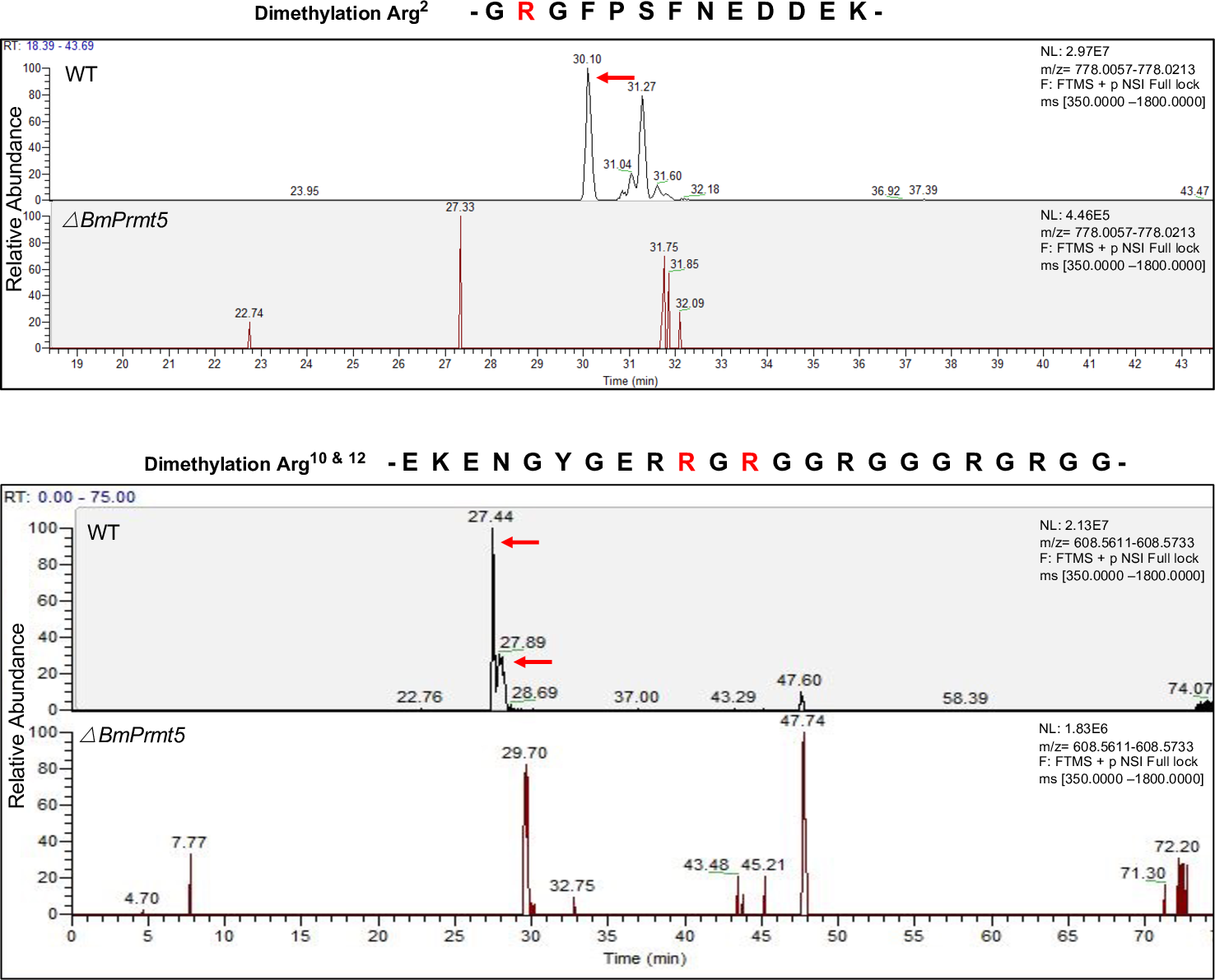

Supplement: S2 Fig — The red arrow represents dimethylated sites of BmVasa peptide. ΔBmPrmt5 represents BmPrmt5 mutants. (TIF) [file pgen.1010600.s003.tif]

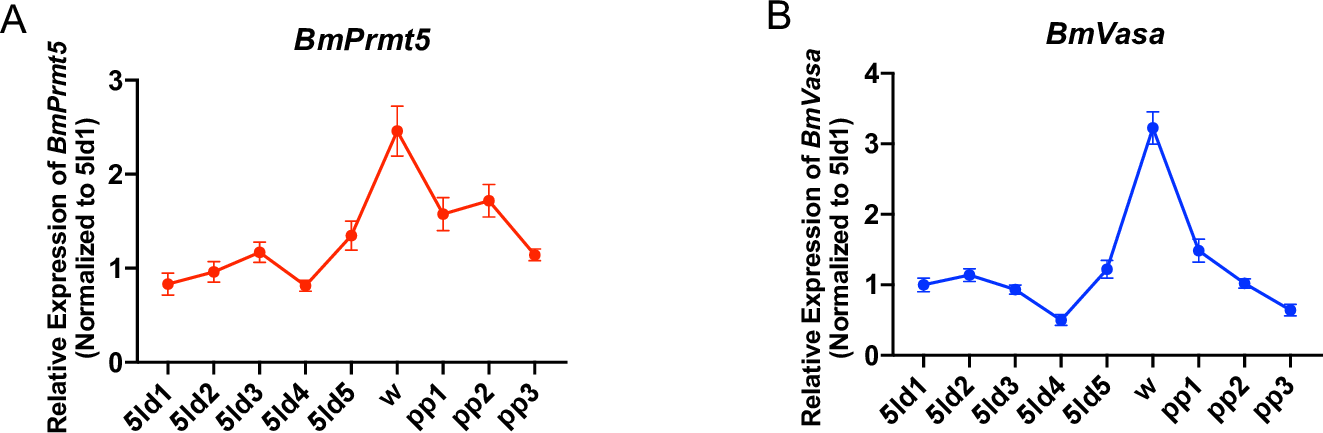

Supplement: S3 Fig — (A and B) qRT-PCR analyses of levels of A) BmPrmt5 mRNA and B) BmVasa in testis from day one of the fifth instar to the third day of the pupal stage. (TIF) [file pgen.1010600.s004.tif]

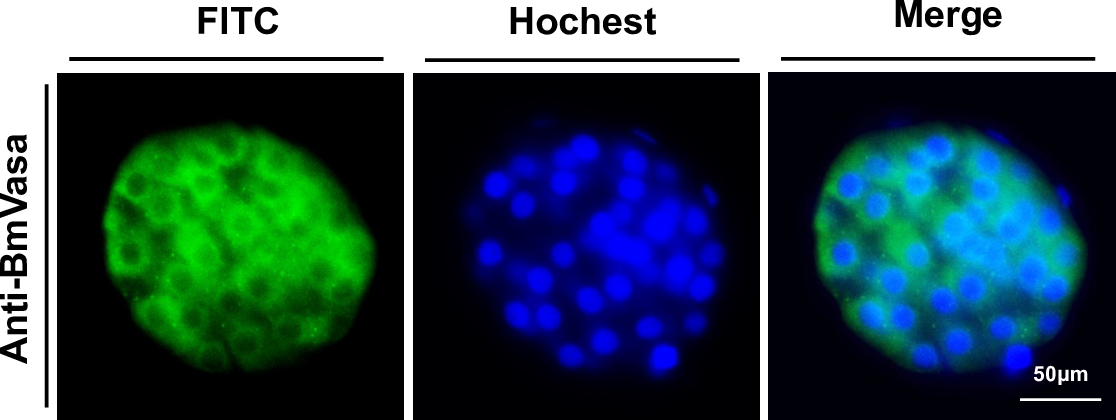

Supplement: S4 Fig — Green, BmVasa; Blue, Hoechst. Scale bar, 50 μm. (TIF) [file pgen.1010600.s005.tif]

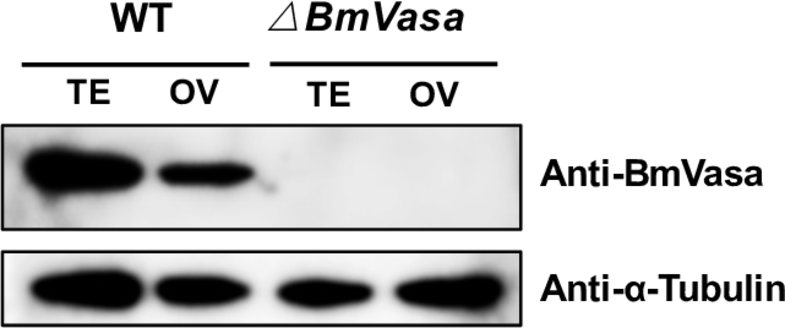

Supplement: S5 Fig — TE and OV denote testis and ovary, respectively. ΔBmVasa represents BmVasa mutants. (TIF) [file pgen.1010600.s006.tif]

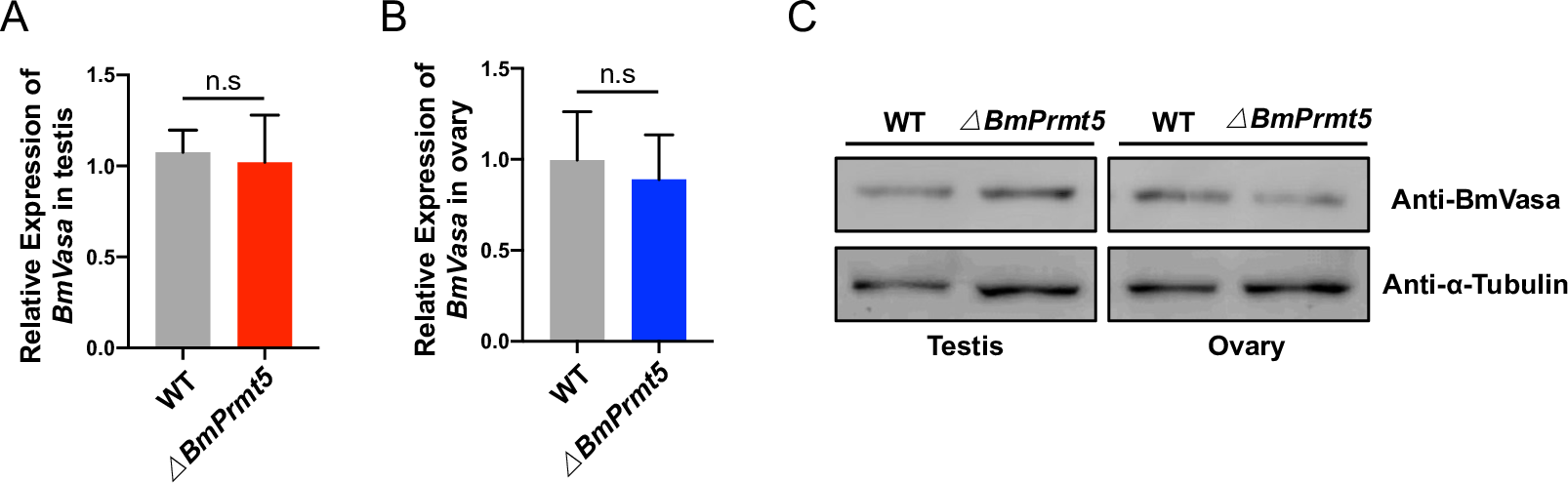

Supplement: S6 Fig — (A and B) qRT-PCR analysis of BmVasa transcript in BmPrmt5 mutant. A) testis and B) ovary (C) Western blot analysis of BmVasa protein levels in testis and ovary of BmPrmt5 mutants. ΔBmPrmt5 represents BmPrmt5 mutants. (TIF) [file pgen.1010600.s007.tif]

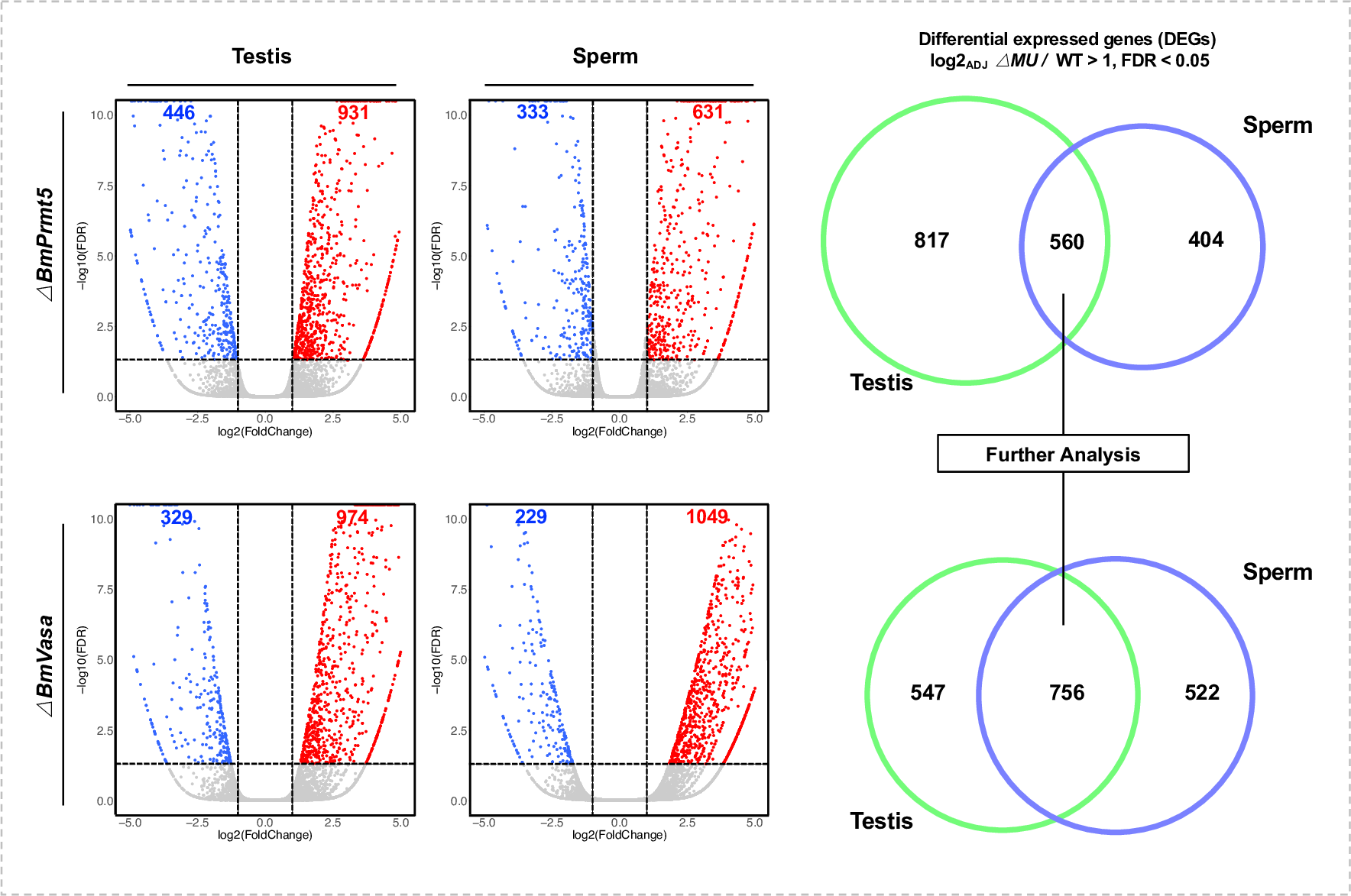

Supplement: S7 Fig — Left: Volcano plots of DEGs in BmPrmt5 and BmVasa mutant testis and sperm samples. Red and blue represent up- and down-regulated DEGs, respectively, with a fold change > 1 and false discovery rate (FDR) < 0.05. Right: Venn diagram of the number of genes commonly and specifically differentially regulated in testis (green) and sperm (blue) samples of BmPrmt5 mutants and BmVasa mutants relative to WT. The “sperm-specific” DEGs were used for further analysis. ΔBmPrmt5 and ΔBmVasa represent BmPrmt5 and BmVasa mutants, respectively. (TIF) [file pgen.1010600.s008.tif]
